# Executive Functions of Six-Year-Old Boys with Normal Birth Weight and Gestational Age

**DOI:** 10.1371/journal.pone.0036502

**Published:** 2012-04-30

**Authors:** Desiree Yee-Ling Phua, Anne Rifkin-Graboi, Seang-Mei Saw, Michael J. Meaney, Anqi Qiu

**Affiliations:** 1 Singapore Institute for Clinical Sciences, The Agency for Science, Technology and Research, Singapore, Singapore; 2 Singapore Eye Research Institute, Singapore, Singapore; 3 Department of Epidemiology and Public Health, National University of Singapore, Singapore, Singapore; 4 Sackler Program for Epigenetics and Psychobiology at McGill University, Douglas Mental Health University Institute, Montreal, Canada; 5 Department of Bioengineering, National University of Singapore, Singapore, Singapore; 6 Clinical Imaging Research Centre, National University of Singapore, Singapore, Singapore; Centre Hospitalier Le Vinatier (Bât. 452), France

## Abstract

Impaired fetal development, reflected by low birth weight or prematurity, predicts an increased risk for psychopathology, especially attention deficit hyperactivity disorder (ADHD). Such effects cut across the normal range of birth weight and gestation. Despite the strength of existing epidemiological data, cognitive pathways that link fetal development to mental health are largely unknown. In this study we examined the relation of birth weight (>2500 g) and gestational age (37–41 weeks) within the normal range with specific executive functions in 195 Singaporean six-year-old boys of Chinese ethnicity. Birth weight adjusted for gestational age was used as indicator of fetal growth while gestational age was indicative of fetal maturity. Linear regression revealed that increased fetal growth within the normal range is associated with an improved ability to learn rules during the intra/extra-dimensional shift task and to retain visual information for short period of time during the delayed matching to sample task. Moreover, faster and consistent reaction times during the stop-signal task were observed among boys born at term, but with higher gestational age. Hence, even among boys born at term with normal birth weight, variations in fetal growth and maturity showed distinct effects on specific executive functions.

## Introduction

Impaired fetal development, reflected by low birth weight or prematurity, adversely affects cognitive development, with implications for academic [1], [2], [3], [4] and behavioral problems [5], [6]. Past research focuses primarily on premature (i.e., <32 weeks gestation) children or those born small-for-gestational-age (i.e., <2500 g). Only recently have the effects of variation in the quality of fetal development across the normal population garnered attention. Since more than 80% of births in developed countries are full-term (i.e., ≥37 weeks) and within the normal birth weight range (>2500 g) [7], [8], it is important to consider the broader relation between fetal development and cognitive functioning. Indeed, Yang et al. [9] observed a positive association between gestational age and intelligence quotient (IQ) among children born at term with normal birth weight. Furthermore, population-based studies examining both variation in birth weight and risk for disorders such as attention deficit hyperactivity disorder (ADHD) [10] underscore the potential importance of subtle variation in fetal growth.

Variations in fetal growth and maturity within the normal range predicts cognitive abilities as measured by IQ [11], verbal reasoning, and math testing [9], [12], [13]. While these global cognitive measures independently relate to birth weight [12], [13], [14], [15] and gestational age [2], [9], few studies have examined both fetal growth *and* maturity on specific forms of cognition. Such studies are essential to moving beyond the broad relations between fetal risk and global outcomes. Indeed, pathways by which fetal development influences risks for specific cognitive and emotional disorders, such as ADHD, remain unknown. Global cognitive measures provide little indication of the effects of fetal development on specific functional domains. A precise definition of those forms of cognitive function that associate with variation in fetal development is essential to translating epidemiological findings into clinical models.

To our knowledge this study is the first to examine variation in fetal growth (i.e., birth weight), maturity (i.e., gestational age), and executive functioning among six-year-old boys born at term across the normal birth weight range. Executive functions refer to cognitive processes required for attention, working memory and behavioral regulation, and are implicated in behavioral problems [16], [17] and academic achievement [18], [19]. We anticipated that variations in fetal growth and maturity across the normal population would result in relatively *subtle* differences in specific functions; thus we focused upon six-year-old boys to avoid possible heterogeneous gender or age outcomes. Previous studies demonstrate males are more susceptible to adverse prenatal circumstances [20], [21] and exhibit strong association of prenatal circumstances to general intelligence [11] and visual attention [22]. Furthermore, subtle cognitive differences are likely to be more consequential at this age as formal schooling requires children to adjust to new experiences and greater cognitive and behavioral demands [2].

## Methods

### Participants

One-hundred and ninty-five ethnically Chinese, Singaporean boys (*range* = 72–84 months, *M* = 78.3 months, *SD* = 3.7 months) were recruited from nine primary schools and an existing cohort study [23]. Written informed consent was obtained from parents. The Institutional Review Board of the National University of Singapore specifically approved this study.

**Table 1:** Multiple regression coefficients for effects of birth weight and gestational age on executive functions of boys at age 6.

Task	Measures	Birth Weight		Gestational Age	
		*β*	*p*	*β*	*p*
IED	Stage 1 Errors	−.21	.004	.003	.97
	Pre-EDS errors	−.02	.84	−.04	.58
	EDS errors	−.15	.04	.03	.68
	Stages completed^a^	.08	.25	−.07	.33
SST	Mean reaction time	−.08	.26	−.21	.004
	Reaction time standard deviation	−.13	.06	−.22	.002
	Stop-signal reaction time	−.09	.22	−.11	.13
DMS	Total correct (12 seconds)	.20	.01	.07	.32
SWM	Between search errors (6 boxes)	.09	.21	−.01	.89
	Strategy	.01	.87	−.08	.28

*Note.* IED = intra/extra-dimensional shift task; EDS = extra-dimensional shift; SST = stop-signal task; DMS = delayed-matching-to-sample task; SWM = spatial working memory task.

a Logistic regression used instead of multiple linear regression.

Birth parameters were obtained from the health booklets, which reliably document birth outcomes by the hospital physician present at birth. All participants were born at 37 to 41 weeks (*M* = 38.9 weeks, *SD* = 1.1 weeks) with birth weight between 2530 to 4110 g (*M* = 3231.3 g, *SD* = 357.2 g). Gestational age was calculated from mother's last menstrual date and confirmed by first trimester crown rump length using ultrasonography. Exclusion criteria included adverse prenatal indicators (e.g., maternal smoking or alcoholism, gestational complications), and chronic medical or mental conditions in potential participants, which was obtained from a parental report. The family's socioeconomic status (monthly household income) was obtained from survey questionnaires conducted as a part of a scheduled appointment. The family's social economic status (SES) was categorized into four categories according to the monthly household income (less than S$1000; S$1000∼S$2999; S$3000∼S$4999; S$5000 and above). In this study, there was no family with monthly income below S$1000. There were 8.4% of the families with monthly income between S$1000 and S$2999, 27.7% of the families with monthly income between S$3000 and S$4999, 63.9% of the families with monthly income above S$5000.

### Measures of executive functions

The Cambridge Neuropsychological Test of Automated Battery (CANTAB) includes language-independent cognitive tests [24] administered on a computer fitted with a touch-sensitive screen and 2-button response pad. Participants were first screened with two motor and learning tasks to verify the ability to follow simple instructions. Subsequently, participants performed the following tasks: intra-/extra-dimensional shift (IED), stop-signal task (SST), delayed-matching-to-sample task (DMS), and spatial working memory (SWM).

Intra/extra-dimensional shift (IED). The IED is a test of rule learning and cognitive flexibility. In each trial participants were shown two abstract images, each comprised of a shape and an overlapping line. They were instructed to choose the correct image in accordance with an underlying rule (e.g., for the first stage the subjects were always required to respond to the shape of the image; see [25] for detailed description). The IED involves a total of nine stages. The number of errors committed on stage 1 indicates proficiency in detecting and learning the implicit rule of the task based on feedback from the experimenter as to whether the choice was correct. Stage 6 involved an intra-dimensional shift where shape remains the target cue, but the ‘correct’ shape changes. Stage 8 was the extra-dimensional shift (EDS stage) where participants must learn to shift attention from the previously correct dimension (the shape of the stimulus) to the newly correct dimension (the line). The number of errors made at the EDS stage indicates proficiency in extra-dimensional set-shifting. The total number of errors from stages 1 to 7 is referred to as Pre-EDS errors and indicates proficiency in maintaining selective attention. Successful completion of stages indicates ability to maintain attention and the flexibly to shift in response to the demands of the task.

#### Stop-signal task (SST)

Based on the race-model and stop-signal paradigm, the SST is commonly used to assess response inhibition. Participants were instructed to withhold their response to the Go stimuli (an arrow on the screen) whenever they heard the stop signal (a beep tone). The mean reaction time (MRT) is used as the measure of the speed to respond to the arrow stimuli on the screen. The standard deviation of the reaction time (SDRT) indicates the variation in reaction times over the task. The stop-signal reaction time (SSRT) is the measure of the ability to inhibit the response to the arrow.

#### Delayed matching to sample (DMS)

The DMS is a test of visual working memory. In each trial a complex visual pattern (the sample) was briefly shown. The sample was then covered and participants saw four patterns below the sample after a delay of 0 s, 4 s or 12 s. Subjects were told to select the pattern identical to the sample. The number of correct answers is used as the measure of visual working memory. Only analyses of 12 s trials were reported in this study as the outcomes for trials with shorter delays revealed ceiling effects.

#### Spatial working memory (SWM)

The SWM is a self-ordered searching task that required participants to maintain and update spatial information in working memory. Participants were required to search through six boxes for blue ‘tokens’. Only one token is hidden at one time and there are six tokens to be found on each trial. Participants were specifically told not to return to the same boxes where a blue token had been previously found as the token would never be hidden in the same box. A between-search error is scored when participants return to a box where a token has already been found and is used as the measure for spatial working memory.

To reduce the load on the working memory, participants could use the strategy of searching using a pre-determined sequence [26], [27]. In other words, when participants found a token, they could restart the search using the sequence they previously used. The strategy score estimates the extent that this strategy is employed and gives an indication of the participant's strategic thinking ability.

### Statistical analysis

To determine birth weight adjusted for gestational age (BW), the association between birth weight and gestational age was assessed in the larger Singaporean cohort (*n* = 1523, gestational age: 37 to 41 weeks; birth weight: >2500 g; Dirani, et al., 2010) using linear regression with the mean-centered gestational age as a main factor (BW∼β (GA-mean(GA))+residual). The residual was defined as BW that reflects relative fetal growth and can be considered statistically independent from gestational age.

In contrast to what might be appropriate for studies using the wider distribution of birth weight and maturity [28], [29], within our range-restricted study, statistical models assumed a linear relationship between executive functions and fetal development. A linear regression model with BW and GA as main factors was used to examine effects on cognitive performance. Age at the time of testing served as a covariate. Multiple comparisons were corrected using a Bonferroni method for individual cognitive tasks (i.e., the level of significance was determined as 0.05 divided by the number of linear regression models performed for one cognitive task).

As SES can be a potential cofounding factor, influences of SES on adjusted BW and GA as well as cognitive measures were examined using one-way ANOVA. No SES group differences were found in adjusted BW (*p* = .550), GA (*p* = .967), and all the measures of the abovementioned executive functions (*p*>.20). Hence, SES was not further considered as covariate in the linear regression analysis on the relationship of fetal development with executive functions.

## Results

### Intra/Extra-Dimensional Shift (IED)

**Figure 1:**
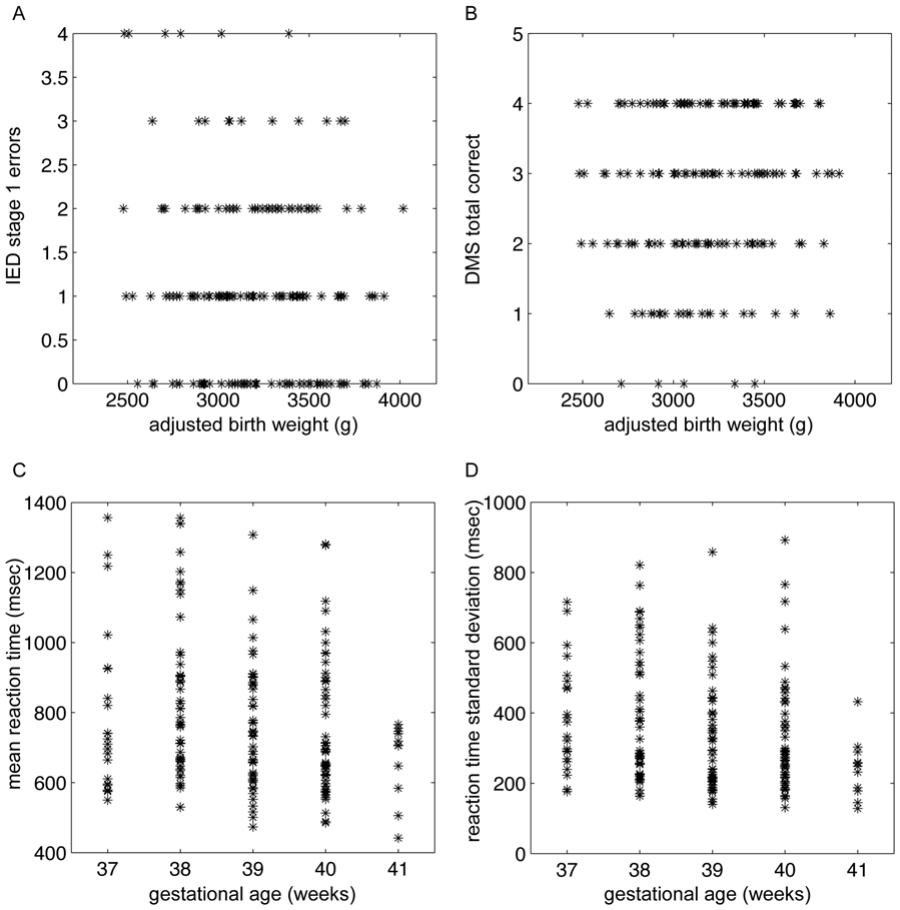
Scatter plots of executive functions with adjusted birth weight and gestational age. Abbreviations: IED = intra/extra-dimensional shift task; SST = stop-signal task; DMS = delayed-matching-to-sample task.

Seven boys (3.6%) completed only stage 1 of the task and the data from these participants were removed from the IED analyses. There was a significant effect of BW on the number of errors made in stage 1 (*p* = .004) (Table 1, Figure 1A), suggesting that children with lower BW were slower in detecting and learning the rule, thus committing more stage 1 errors. There were no significant GA effects.

There were no significant effects of BW (*p* = .84) or GA (*p* = .58) on Pre-EDS errors. Also, there were no significant effects of BW (*p* = .04) or GA (*p* = .68) on EDS errors after the correction of multiple comparisons. Successful completion of all nine stages reflects ability to discern the rule based on feedback, maintain selective attention and to shift attention within and beyond a dimension of the visual stimuli when required. 81 boys (41.5%) completed the task, 114 boys (58.5%) did not manage to complete all the stages. We used logistic regression to examine the effect of BW and GA on the odds of successfully completing all the IED stages. Neither BW nor GA significantly increased the odds of completion (BW: AOR = 1.00, Wald's χ^2^(1) = 0.27, *p* = .25; GA: AOR = 0.94, Wald's χ^2^(1) = 0.25, *p* = .33). There were no differences in BW (*t*_193_ = −0.47, *p* = .64) nor GA (*t*_193_ = 0.46, *p* = .65) between the boys who were or were not able to complete all the stages.

### Stop-signal task (SST)

There were significant GA effects in the reaction time measures. Lower GA was significantly associated with higher mean (*p* = .004, Table 1) (Figure 1C) and standard deviation (*p* = .002, Table 1) (Figure 1D) of reaction times, thus indicating slower and more varied response speeds. There were no significant BW effects on either measure.

Neither BW nor GA was significantly associated with response inhibition, as measured by SSRT (*p* = .22 and .13 respectively).

### Delayed matching-to-sample (DMS)

There was significant BW effect on the number of correct recalls after 12 seconds delay (*p* = .01, Table 1) (Figure 1B), with higher BW associated with higher number of correct recalls. There was no significant GA effect (*p* = .32).

### Spatial working memory (SWM)

Neither BW nor GA was significantly associated with the between search errors (BW: *p* = .21; GA: *p* = .89) and strategy (BW: *p* = .87; GA: *p* = .28).

## Discussion

We examined the effects of fetal growth (i.e., BW) and maturity (i.e., GA) within the normal range on executive functioning in six-year-old boys. Our results revealed distinct effects of fetal growth and gestational age on executive functioning. Fetal growth was related to the IED measures of rule acquisition as well as DMS measure of visual short-term memory; fetal maturity was associated with the SST response measures of speed and variation.

Our findings suggest that increased fetal growth within the normal range is associated with an improved ability to learn rules without explicit instruction [30], and to retain visual information for short period of time. This effect may account for the varied outcomes reported in previous studies in mathematics [31], complex visual tasks [32] and reading comprehension [33], each of which partially relies upon implicit rule learning and short-term visual memory. General deficits in implicit learning, may affect the acquisition of domain-specific (e.g., mathematical or linguistic) non-declarative knowledge, which in turn manifest in poorer performance within these domains [34]. Similarly, deficits in visual memory may affect a host of academic outcomes [35]. As such, children with birth weight at the lower end of the normal range who experience greater difficulties in both implicit learning and visual short-term memory may perform relatively poorly in subjects like reading or mathematics, which rely heavily upon these cognitive functions [36].

In addition to fetal growth, fetal maturity also exerted independent effects on cognitive development. Premature exposure to the extra-uterine environment, even by one or two weeks, might constrain neuronal development [37], [38], [39] due to the rapid brain growth during the final weeks of gestation [40]. Slower and inconsistent reaction times during the stop-signal task were observed among boys born at term, but with lower GA. Slower and more variable reaction times are characteristic of many neurodevelopmental disorders, including ADHD [37], [38], [39]. Reaction speed and variability may relate to cognitive processing speed [39], attentional resources [38] or intelligence [41]. A similar association was reported by Yang et al. [9] who found that even among children born at term, GA is positively associated with IQ scores. Relative shorter gestation duration within the normal range was also found to relate to infant neuromotor development [29]; such effects might sustain to middle childhood thus accounting for the effects on response times in our study.

Differential effects of fetal growth and maturity on cognitive functions are consistent with results demonstrating BW effect on total brain volume, but GA effect on regional brain volumes [42]. Importantly, within our sample of healthy boys born at term with normal BW, we identified executive functions as a function of fetal growth and maturity. These findings support the idea that fetal development needs to be examined on a continuum [43] and the influences are not limited to extreme ranges but occur across the entire population. Nevertheless, this may not be generalizable to girls as suggested by previous findings of gender differences in fetal development and its relation with academic performance [44].

A potential limitation of the study is the lack of the inclusion of covariates. While environmental factors, such as parental education [28], or parity [45] might weaken the association between fetal development and subsequent cognition, they do not invalidate the association. Our study involved the families above the poverty level and hence did not find any association of socioeconomic status with the birth outcomes and any executive function. This is consistent with previous evidence showing socioeconomic status may weaken pre-natal influences on subsequent cognition but fetal development is continually found to exert independent effects on cognitive development [e.g., 31,46,47]. The quality of the fetal environment is likely to mediate relations between the external environment and cognitive development [28]. Additionally, studies of prenatal influences, ours included, use birth outcomes such as weight and gestational age as proxies for the quality of fetal development.

### Conclusion

Reliance on global measures of neural function (e.g., IQ) does not advance our ability to establish causal cognitive pathways that fetal development to specific neurodevelopmental disorders, such as ADHD, nor do they inform on specific cognitive problems associated with executive dysfunction. Our study showed distinctive roles of fetal growth and maturity in executive functioning among boys born with normal birth weight and at term, suggesting population-wide effects of fetal influences. Furthermore, as predicted, relative differences in fetal development did not relate to global dysfunction, i.e., fetal development in this normal sample related to some, but not all, executive functions. This suggests that the majority population born at ‘term’ and within the normal range for birth weight should not be considered a homogenous group. Likewise subtle prenatal influences may have a large societal impact. As such, we should optimize maternal pre-natal health for all women, not only those at risk for preterm deliveries and inter-uterine growth retardation.
